# Decoding the Microbiome’s Influence on Rheumatoid Arthritis

**DOI:** 10.3390/microorganisms11092170

**Published:** 2023-08-28

**Authors:** Donatella Coradduzza, Marco Bo, Antonella Congiargiu, Emanuela Azara, Maria Rosaria De Miglio, Gian Luca Erre, Ciriaco Carru

**Affiliations:** 1 Department of Biomedical Sciences, University of Sassari, 07100 Sassari, Italy; m.bo4@studenti.uniss.it (M.B.); antcong895@gmail.com (A.C.); 2 Institute of Biomolecular Chemistry, National Research Council, 07100 Sassari, Italy; e.azara@icb.cnr.it; 3 Department of Medicine, Surgery and Pharmacy, University of Sassari, 07100 Sassari, Italy; demiglio@uniss.it (M.R.D.M.); glerre@uniss.it (G.L.E.); 4 Control Quality Unit, Azienda-Ospedaliera Universitaria (AOU), 07100 Sassari, Italy

**Keywords:** biomarkers, microbiome, rheumatology, rheumatoid arthritis, autoimmune disease

## Abstract

The aim is better to understand and critically explore and present the available data from observational studies on the pathogenetic role of the microbiome in the development of rheumatoid arthritis (RA). The electronic databases PubMed, Scopus, and Web of Science were screened for the relevant literature published in the last ten years. The primary outcomes investigated included the influence of the gut microbiome on the pathogenesis and development of rheumatoid arthritis, exploring the changes in microbiota diversity and relative abundance of microbial taxa in individuals with RA and healthy controls (HCs). The risk of bias in the included literature was assessed using the GRADE criteria. Ten observational studies were identified and included in the qualitative assessment. A total of 647 individuals with RA were represented in the literature, in addition to 16 individuals with psoriatic arthritis (PsA) and 247 HCs. The biospecimens comprised fecal samples across all the included literature, with 16S rDNA sequencing representing the primary method of biological analyses. Significant differences were observed in the RA microbiome compared to that of HCs: a decrease in *Faecalibacterium*, *Fusicatenibacter*, *Enterococcus*, and *Megamonas* and increases in *Eggerthellales*, *Collinsella*, *Prevotella copri*, *Klebsiella*, *Escherichia*, *Eisenbergiella*, and *Flavobacterium*. There are significant alterations in the microbiome of individuals with RA compared to HCs. This includes an increase in *Prevotella copri* and *Lactobacillus* and reductions in *Collinsella*. Collectively, these alterations are proposed to induce inflammatory responses and degrade the integrity of the intestinal barrier; however, further studies are needed to confirm this relationship.

## 1. Introduction

The gut microbiome has gained vast attention in recent years due to its emerging importance in human health and disease [1,2], with evidence demonstrating its influence in the development of autoimmune diseases [3,4]. The microbiome comprises bacteria, archaea, viruses, and eukaryotes and has collectively been implicated in contributing to the development of autoimmune diseases through the pro-inflammatory and immune pathways [5,6,7]. Like many diseases [8], rheumatoid arthritis (RA) arises from a combination of genetic and epigenetic components [9]. However, environmental factors also play a crucial role [10], such as exposure to cigarette smoke, dust, and particularly the microbiome, which represents an “internal” environment.

Rheumatoid arthritis is an autoimmune disease characterized by chronic inflammation of the joints, leading to pain, swelling, stiffness, and potential damage in the synovial joints. It represents one of the most prevalent autoimmune diseases, affecting an estimated 1% of the global population, and is concurrent with numerous comorbidities, such as cardiovascular, pulmonary, psychological, and musculoskeletal disorders [11,12]. Genome-wide association studies have demonstrated the contribution of genetic factors in RA susceptibility, such as genes in the major histocompatibility complex (MHC) [13]. However, research into disease presentation in monozygotic twins and the low concordance of RA amongst these populations suggest the involvement of environmental factors in the development of RA [14,15,16]. An essential interaction exists between the components of the adaptive and innate immune systems [17,18].

While the exact cause of RA is still not fully comprehended, research suggests that both genetic and environmental factors contribute to its development [19]. The gut microbiome, in particular, has been a subject of interest in relation to autoimmune diseases, including RA [6]. As described by Tsetseri et al., the gut microbiota and the immune system have a close bidirectional relationship, widely established to play a significant role in several systemic autoimmune diseases [20]. Ongoing research is investigating alternative strategies involving the microbiome that may also have important implications, with rapid growth in this area of study. Furthermore, previous studies [21] have suggested a potential involvement of chronic triggers of the gut microbiome, while responses to fasting have been examined as well [21,22,23].

The gut microbiota can influence the immune system and inflammation [24,25,26,27,28,29,30], and imbalances in the gut microbial communities have been observed in individuals with RA [31,32]. A recent meta-analysis indicated that gut dysbiosis is prominent in RA patients and is characterized by a reduction in anti-inflammatory butyrate-producing bacteria, such as *Faecalibacterium*, and augmentation of pro-inflammatory bacteria, such as *Streptococcus* [33]. Moreover, dysbiosis of the gut microbiota contributes to the onset and progression of the disease through multiple pathways, including the production of pro-inflammatory cytokines, promotion of Th17 cell differentiation, and modulation of regulatory T cell function [34,35]. In addition, additional studies have identified correlations between the early stages of rheumatoid arthritis (RA) and a recently discovered species of bacteria in the gut microbiome known as *Subdoligranulum didolesgii* [36,37]. This bacterium was found in the feces of four out of twenty-four individuals who were either at risk for or had been diagnosed with RA, but it was not detected in the feces of twelve healthy individuals. Importantly, this bacterial species has been recognized as a trigger for the development of autoantibodies that target body tissues. The hypothesis suggests that a mucosal immune response to *S. didolesgii* in the gut could potentially lead to a systemic immune response throughout the body [38].

Similar findings have also been observed for Sjogren’s syndrome and systemic lupus erythematosus, suggesting the contribution of gut microbiota dysbiosis in the occurrence or development of RA [33]. The current literature proposes that gut dysbiosis precedes arthritis and induced local intestinal inflammation. As a result, systematic inflammation occurs in genetically predisposed individuals, contributing to the development and progression of RA [20]. 

Researchers are also investigating the potential therapeutic applications of manipulating the gut microbiome in treating or managing autoimmune diseases like RA. Some studies have explored the use of probiotics, prebiotics, or fecal microbiota transplantation (FMT) as potential interventions [39]. Collectively, these interventions target the three proposed mechanisms by which the microbiome contributes to RA pathogenesis, including inflammatory responses (*Prevotella copri* and *Lactobacillus*), molecular mimicry (*Prevotella copri*), and loss of intestinal barrier integrity (*Collinsella*) [20]. Probiotics demonstrated their ability to modulate the gut microbiota, reduce inflammation, and alleviate symptoms in animal models of arthritis. The presence of gut dysbiosis may contribute to the immune dysfunction characteristic of RA, making the modulation of the gut microbiota a potential therapeutic approach. Moreover, the data support the potential use of gut microbiota-related biomarkers in diagnosing and predicting the prognosis of RA [16].

A significant study was undertaken to establish a connection between the gut microbiome and the prognosis of rheumatoid arthritis (RA). This breakthrough research unveils the potential to foresee a patient’s future RA prognosis through the intricate analysis of trillions of microorganisms, including bacteria, viruses, and fungi residing in their gastrointestinal tract. Employing a comprehensive genomic analysis, the researchers pinpointed several distinctive traits within the gut microbiome that bear relevance to the patient’s forthcoming prognosis. To further enhance the accuracy of predictions, they harnessed the power of artificial intelligence (AI) and deep learning. Remarkably, this AI-driven approach achieved an impressive accuracy rate of 90 percent in forecasting clinical improvement. The convergence of data from the gut microbiome with cutting-edge AI technology opens up new avenues for prognosticating the trajectory of RA with heightened precision [40,41].

Promising advancements in the management of RA involve cultivating beneficial changes in gut bacteria through dietary modifications, such as augmenting fiber consumption and incorporating fermented foods. Research endeavors delve into the intricate interplay between dietary practices, nutritional factors, and RA [42,43]. This investigation proposes that strategic dietary interventions, encompassing anti-inflammatory regimens and the integration of omega-3 fatty acids, exhibit potential in alleviating the impact of RA [44,45,46]. Furthermore, the study accentuates substantial disparities in the gut microbiota observed between individuals with RA and their healthy counterparts, underscoring the importance of contemplating constructive alterations in gut microorganisms via dietary interventions [47].

The effects of a probiotic mixture on inflammatory biomarkers and oxidative/nitrosative profiles in subjects with RA were analyzed. The study found that the probiotic mixture, consisting of *Lactobacillus casei*, *Lactobacillus rhamnosus*, *Bifidobacterium bifidum*, and *Bifidobacterium longum*, led to a significant reduction in inflammatory biomarkers and improved the oxidative/nitrosative profile of participants. A meta-analysis further supports the efficacy of probiotic supplementation in RA, indicating a positive effect on disease activity and a trend toward improved lipid profile, insulin sensitivity, and antioxidant markers [48,49].

Similar to the gut microbiome, the oral microbiome may contribute to immune dysregulation and inflammation, thus influencing the pathogenesis of RA. A study involved 87 patients with active RA who were either responding inadequately to conventional disease-modifying synthetic antirheumatic drugs, had severe comorbidities, or were unresponsive to treatment. Researchers analyzed correlations between patients’ disease activity, disease biomarkers, gut bacterial counts, fecal and serum lipopolysaccharide (LPS), LPS-binding protein (LBP), endotoxin neutralizing capacity (ENC), and serum levels of antibodies to antimicrobial substances IgG and IgA. The results suggest that substances in the oral and intestinal microbiota may influence disease activity in RA patients. Additionally, clinical association studies indicate that periodontal treatment, which includes oral hygiene and supragingival scaling, reduced DAS28-CRP scores in patients with RA, further supporting the potential pathogenic role of microbial infection in RA [37,50].

This area of research is novel, and more rigorous clinical trials are needed to determine the safety and efficacy of RA treatments targeting the microbiome. Furthermore, additional investigations are required to establish causality between the microbiome and the development of rheumatic diseases and identify specific bacterial strains that contribute to its pathology [51,52,53]. This review critically explores and presents the available data from observational studies on the pathogenetic role of the microbiome in the development of RA. The molecular mechanisms by which the microbiome may contribute to RA will also be investigated.

A comprehensive search strategy was devised and employed for the literature databases PubMed, Scopus, and Web of Science by an independent author between 20 July and 25 July 2023, covering a span of the most recent decade. The keywords “microbiome”, “gut microbiota”, “gut microbiome”, “RA”, “rheumatoid arthritis”, “inflammation”, “autoimmune”, and “autoimmunity” were used to refine the scope of the literature identified in the initial searches [54,55]. The literature was then evaluated for relevance and was included if it explored and reported on the role of the microbiome and abundance of the microbiota in RA. This comprised its role in the pathology of RA, such as the induction of pro-inflammatory pathways. Eligibility restrictions were also applied, with the literature being excluded if it did not include human participants, were review articles, were published prior to 2013, or were published in any non-English language. The primary outcomes of interest were measures of microbial diversity, namely changes in α-diversity and relative abundance of various taxa. For the purpose of this review, microbiome diversity refers to the variety and abundance of a given bacterial species in a defined unit of study [56,57]. In this study, the relative abundances at the phylum, family, and genus levels were examined. If required and necessary, units were converted so that related outcomes were on consistent scales. The quality of the included literature was evaluated in concordance with GRADE criteria for determining the quality of evidence and recommendation for use [58]. These criteria consider the methodological quality, directness of evidence, heterogeneity, precision of effect estimates, and the risk of publication bias. This yielded a score of high, moderate, or low level of evidence and recommendation for use.

## 2. Relevant Sections

### 2.1. Identification of the Literature 

A preliminary selection of 463 records, determined to be germane to the research, was initially pinpointed in accordance with the applied search strategy. Following the removal of duplicate records in EndNote X9, the titles and abstracts of 184 records were screened against the inclusion and exclusion criteria. At this stage, a total of 156 records were excluded. The remaining 28 records were assessed as full-text articles for eligibility, with 18 being removed as they were either review articles or published in a non-English language. Consequently, ten full-text articles were deemed relevant and subject to data extraction in this qualitative analysis.

### 2.2. Study Characteristics 

All studies included, Table 1, in this review evaluated the relationship between the microbiome and the development of RA. A total of 647 individuals with RA were included, 16 with psoriatic arthritis (PsA) and 247 HCs. The biospecimens comprised fecal samples across all the included literature, with 16S rDNA sequencing representing the primary method of biological analyses (8 studies, 80%). However, one study adopted culture-independent microbiota analysis (1 study, 10%) and another MiSeq sequencing (1 study, 10%).

### 2.3. Relative Abundance

Compared to healthy controls, there were notable differences in the relative abundance of several bacteria in the microbiome. This included a decrease in *Faecalibacterium*, *Fusicatenibacter*, *Enterococcus,* and *Megamonas* and increases in *Eggerthellales*, *Collinsella*, *Prevotella copri*, *Klebsiella*, *Escherichia*, *Eisenbergiella,* and *Flavobacterium*.

Besides *Prevotella copri*, it was also observed that several species belonging to the Prevotella genus increased in the RA microbiome [21]. The microbiome dysbiosis observed in RA patients correlated with altered endotoxin-neutralizing capacity, serum lipopolysaccharide, and anti-Pg-LPS IgA antibody levels [3].

Additional outcomes observed suggest the microbiota of individuals in “pre-clinical RA stages” differs from that of chronic RA patients and HCs [59]. The role of diet was also evaluated, with a healthier gut microbiota composition observed in RA patients who adhered to a Mediterranean diet [66]. 

### 2.4. Quality Appraisal 

Table 2 summarizes the overall risk of bias for each study included in this review and the recommendation for use, with all studies scoring highly. All studies achieved a low risk of bias with regard to methodological quality, heterogeneity, precision of effect estimates, and publication bias. Four studies, however, presented an unclear risk of bias for the directness of evidence [3,42,65,67].

## 3. Discussion

The gut microbiome has garnered significant attention in recent years due to its burgeoning importance in human health and disease. Substantiated by mounting evidence, its involvement in the genesis of autoimmune diseases has become increasingly apparent [1,2]. This microbiome, composed of a diverse array of bacteria, archaea, viruses, and eukaryotes, collectively assumes a role in the emergence of autoimmune conditions through the intricate tapestry of pro-inflammatory and immune pathways [3,4]. This consortium of genomes, thriving within an ecological community of microorganisms, assumes a pivotal function in both the evolution and progression of rheumatoid arthritis (RA). Notably, the gut microbiome emerges as a sentinel, not merely for immune balance but also as an indicator of the host’s overall health. A disruption in the equilibrium between host and microbiome can reverberate across mucosal and systemic immunity, potentially giving rise to an array of inflammatory and autoimmune diseases [39]. 

In this mosaic of health and ailment, rheumatoid arthritis stands as a poignant example, casting a profound shadow over global health. Characterized by persistent joint inflammation and its far-reaching constellation of symptoms, RA embodies a formidable autoimmune adversary, affecting a substantial segment of the population. Amidst the quest to unveil the intricate pathogenesis of RA, microbial factors have assumed a role of increasing significance [25]. Cumulative evidence underscores the interaction between the gut microbiome and autoimmune diseases, spotlighting its potential influence on conditions such as RA. As an integral component of an ecological ensemble, the gut microbiome exercises control over immune harmony while also unveiling insights into the host’s health. Disruptions in this interaction could trigger a cascade of events with implications for both mucosal and systemic immunity, opening doors to the development of inflammatory and autoimmune disorders [25,39,55].

Undoubtedly, the pathogenesis of RA remains an enigma, with its complexity only deepening as research progresses. Microbial factors have emerged as key players, intertwining with intricate mechanisms and raising questions about their role in disease development. 

*Prevotella copri* was of particular interest among the included literature, with Scher et al. reporting a strong correlation between the abundance of this bacterium and disease in new-onset RA patients. The rationale for this was proposed to result from the reduction in *Bacteroides* and loss of beneficial microbes that were observed following an increase in the *Prevotella copri* abundance [60]. Similarly, *Prevotella copri* antibody responses were rarely found in patients with other rheumatic diseases or in HCs [67,68]. Some specific microorganisms, such as *Prevotella copri* and *Lactobacillus,* have been associated with the promotion of inflammatory responses in the intestinal environment. These inflammatory reactions can impact the immune system, contributing to the development and exacerbation of RA. Another important mechanism is molecular mimicry, in which microbial components can resemble those of the host tissue. In particular, *Prevotella copri* has been identified as a microorganism with molecules similar to those of human joint tissues. This similarity can lead the immune system to mistakenly attack the host’s cells as well, causing joint damage. The third mechanism involves the loss of intestinal barrier integrity. The presence of harmful bacteria, such as *Collinsella copri,* can weaken the protective barrier of the intestine. This allows harmful substances to enter the bloodstream and trigger a systemic immune response, which, in turn, may be involved in the development of RA. These three mechanisms, while distinct, may interact in complex ways, amplifying the role of the microbiome in RA pathogenesis. It is important to continue research in this field to fully understand the involvement of the microbiome in the disease and to develop new targeted therapeutic strategies. The findings of this review substantiate this. Chen et al. conducted a significant study that illuminated the relationship between the gut microbiome and RA. Their research revealed an intriguing observation regarding the abundance of *Collinsella* and its correlation with specific metabolic and immunological factors in individuals with RA. They found that higher levels of *Collinsella* were strongly associated with increased levels of two metabolites: alpha-aminoadipic acid and asparagine. These metabolites play essential roles and are linked to inflammation and autoimmune conditions like RA. Moreover, the study by Chen et al. uncovered a compelling connection between the abundance of *Collinsella* and the production of a pro-inflammatory cytokine called IL-17A, which is known to promote inflammation in RA. The interplay between *Collinsella* abundance, alpha-aminoadipic acid, asparagine, and IL-17A production provides valuable insights into the potential mechanisms by which the gut microbiome may contribute to the pathogenesis of RA. These findings suggest that the presence of *Collinsella* in the gut could be involved in promoting an inflammatory environment that may exacerbate RA symptoms. Overall, Chen et al.’s research offers valuable information on how the gut microbiome’s composition and its interactions with specific metabolites and immune factors could influence the development and progression of RA. Understanding these connections may pave the way for targeted therapeutic approaches aimed at modulating the gut microbiome to manage RA effectively. *Collinsella*’s significant role in altering gut permeability in RA has garnered increasing attention in recent research. The gut barrier serves as a critical defense mechanism, regulating the passage of substances from the gut lumen into the bloodstream. When this barrier’s integrity is compromised, harmful substances, including bacterial components and toxins, can leak into the bloodstream, triggering an immune response and potentially contributing to the development or worsening of autoimmune diseases like RA. Several studies have demonstrated a correlation between higher levels of *Collinsella* in the gut and increased gut permeability in individuals with RA. This bacterium appears to play a part in the breakdown of the intestinal barrier, allowing the translocation of inflammatory agents and other immune-activating molecules into the bloodstream. One potential mechanism through which *Collinsella* may impact gut permeability is by producing metabolites or molecules that disrupt the tight junctions between intestinal epithelial cells. Tight junctions are vital protein complexes responsible for maintaining the integrity of the gut barrier. When these junctions become compromised, the gut becomes “leaky”, facilitating the passage of substances more easily. The increased permeability of the gut can lead to systemic inflammation, as the immune system recognizes the leaked substances as foreign invaders and initiates an immune response. This ongoing inflammatory process may contribute to the chronic inflammation observed in RA and exacerbate the disease symptoms. Understanding *Collinsella*’s role in altering gut permeability offers valuable insights into the complex interactions between the gut microbiome and the pathogenesis of RA. These findings underscore the importance of maintaining a balanced gut microbiome to preserve gut barrier integrity and potentially mitigate the inflammatory processes associated with RA. Future research in this area may explore targeted interventions aimed at modulating *Collinsella* levels in the gut or improving gut barrier function as a potential therapeutic strategy for managing RA and other autoimmune conditions. By gaining a deeper understanding of how specific gut bacteria, like *Collinsella*, influence gut permeability and inflammation, we can advance our understanding of RA’s underlying mechanisms and develop more personalized and effective treatments for patients with this debilitating disease. 

Likewise, the presence of *Prevotella copri* has emerged as a notable factor associated with the majority of new-onset RA patients. Studies and research have indicated a strong correlation between the abundance of *Prevotella copri* in the gut and the onset of RA in previously healthy individuals. This finding suggested that *Prevotella copri* may play a contributory role in triggering or promoting the autoimmune response in genetically susceptible individuals through several hypotheses that have been proposed. One possibility is that *Prevotella copri* may directly interact with the immune system, leading to the production of pro-inflammatory cytokines and activation of immune cells that target joint tissues, resulting in the characteristic inflammation and joint damage seen in RA. Additionally, *Prevotella copri* might also induce molecular mimicry, wherein its components resemble self-antigens found in joint tissues, leading to the immune system mistakenly attacking its host’s joint tissues, further fueling the inflammatory response. 

Furthermore, it is believed that *Prevotella copri*’s presence in the gut may lead to alterations in the gut microbiome composition, disrupting the delicate balance of beneficial and harmful bacteria and promoting an environment conducive to inflammation and autoimmunity for RA development. However, *Prevotella copri*’s association with RA is intriguing and may offer novel strategies for improving the diagnosis and treatment of this debilitating autoimmune disease [18]. 

Regarding *Lactobacillus*, it was observed that this bacterium was significantly more abundant in HCs compared to patients with RA, highlighting the potential role of this specific bacterium in maintaining gut health and immune homeostasis. *Lactobacillus* is a beneficial and probiotic bacterium known for its various health-promoting properties. It modulates immune responses and inhibits the growth of harmful bacteria, helping to maintain the balance of the gut microbiome and contributing to overall well-being. The reduced abundance of *Lactobacillus* in patients with RA raises interesting questions about its potential involvement in the disease. Several hypotheses have been proposed to explain this association as the lower levels of *Lactobacillus* in RA patients might indicate a state of gut dysbiosis, where there is an imbalance in the gut microbial community. This dysbiosis could contribute to an environment that favors inflammation and immune dysfunction, which are characteristic features of RA. Moreover, *Lactobacillus* is known for its immunomodulatory effects, promoting the generation of anti-inflammatory responses and regulatory T cells. The reduced levels of *Lactobacillus* in RA patients may result in an imbalance of immune responses, potentially contributing to the autoimmune process in RA. Furthermore, Lactobacillus is involved in maintaining gut barrier integrity, preventing the translocation of harmful substances from the gut into the bloodstream.

## 4. Conclusions and Future Directions

In this comprehensive review, we present compelling evidence supporting the immune relevance of *Collinsella, Lactobacillus*, and *Prevotella copri* in the context of RA. These novel insights hold significant implications for both the diagnosis and treatment of RA. By thoroughly collating research findings, we have identified these specific microbial players as potential key contributors to the immune dysregulation observed in RA. Their involvement in the disease process opens up valuable opportunities for advancing diagnostic approaches, enabling more accurate and early detection of RA.

Furthermore, gaining an understanding of the immune significance of *Collinsella, Lactobacillus*, and *Prevotella copri* paves the way for targeted therapeutic interventions. The ability to manipulate the gut microbiome and its interactions with the immune system offers promising avenues for developing novel and more effective treatments for RA, with the aim of restoring immune balance and reducing inflammation. Our work not only contributes to advancing the field’s knowledge but also sheds light on potential advancements in precision medicine approaches for managing RA. The implications of our findings extend to personalized treatment strategies that consider individual gut microbial profiles, leading to more tailored and efficient therapies. Moreover, beyond the role of the microbiome and individual bacteria, our review highlights the importance of amino acid pathways in the development of RA. Depletion of L-arginine and aromatic amino acids in RA patients, along with dysregulation of pathways such as tryptophan metabolism, alpha-linolenic acid metabolism, and glycerophospholipid metabolism, may further contribute to the pathogenesis of RA. However, further research is needed to explore the biological roles of these pathways and validate these findings in independent RA populations.

In summary, our review solidifies the role of a probiotic imbalance in both the active and inactive stages of RA. Notably, the evidence presented substantiates the significance of *Collinsella*, *Prevotella copri*, and *Lactobacillus* in the pathogenesis of RA. Nonetheless, unraveling the precise functions of the microbiome in the development of RA warrants further investigation. As we continue to delve into the complexities of the gut microbiome and its impact on autoimmune diseases like RA, we move towards a future where our growing understanding of these microbial interactions holds the potential to revolutionize patient care and outcomes. The exploration of targeted therapies and precision medicine approaches offers hope for improved management and better quality of life for individuals living with RA. 

## Figures and Tables

**Table 1:** Data extraction and summary of the human studies investigating the relationships between gut microbiota dysbiosis and RA.

Author	Study Design	Study Subjects	Biospecimen	Microbiological Analysis	Findings
Chen et al. (2016) [21]	Cross-sectional	*n* = 40 RA *n =* 32 HCs	Fecal samples	16S rDNA sequencing	Decrease in *Faecalibacterium* and expansion of *Eggerthellales* and *Collinsella*.
Scher et al. 2013 [59]	Cross-sectional	*n =* 44 new onset RA *n =* 26 chronic RA *n =* 16 PsA *n =* 28 HCs	Fecal samples	16S rDNA sequencing	*Prevotella copri* strongly correlated with disease in new onset RA patients. Increases in *Prevotella* abundance correlated with a reduction in *Bacteroides* and a loss of reportedly beneficial microbes in new onset RA subjects.
Pianta et al. (2018) [60]	Observational	*n =* 127 RA	Fecal samples	16S rDNA sequencing	*Prevotella copri* antibody responses were rarely found in patients with other rheumatic diseases or in HCs.
Yu et al. (2021) [61]	Cross-sectional	*n =* 26 RA *n =* 26 HCs	Fecal samples	16S rDNA sequencing	*Klebsiella*, *Escherichia*, *Eisenbergiella*, and *Flavobacterium* were more abundant in the RA patients, while *Fusicatenibacter*, *Megamonas,* and *Enterococcus* were more abundant in the HCs.
Kishikawa et al. (2020) [62]	Cross-sectional	*n =* 82 RA *n =* 42 HCs	Fecal samples	16S rDNA sequencing	Multiple species belonging to the *Prevotella* genus increased in the RA gut metagenome.
Alpizar-Rodriguez et al. (2019) [63]	Cross-sectional	*n =* 50 HCs *n =* 83 pre-clinical RA	Fecal samples	Culture-independent microbiota analyses	The microbiota of individuals in “pre-clinical RA stages” was significantly altered compared with FDR controls. A significant enrichment of the bacterial family Prevotellaceae, particularly *Prevotella* spp., in the “pre-clinical RA” group (*p* = 0.04) was observed.
Chen et al. (2021) [64]	Cross-sectional	*n =* 29 RA *n =* 30 HCs	Fecal samples	16s rRNA sequencing	At the genus level, *Bacteroides*, *Faecalibacterium*, and some probiotics decreased in the RA group, while 97 genera, including *Lactobacillus*, *Streptococcus*, and *Akkermansia*, increased in the RA group.
Diamanti et al. (2020) [65]	Cross-sectional	*n =* 30 RA with high adherence to Mediterranean diet, *n =* 30 RA with low adherence to Mediterranean diet	Fecal samples	16s rRNA sequencing	A healthier gut microbiota composition was observed in the high adherence to the Mediterranean diet group, with a significant decrease in *Lactobacillaceae* and an almost complete absence of *Prevotella copri* with respect to the low/moderate adherence group.
Sun et al. (2022) [66]	Cross-sectional	*n =* 42 RA *n =* 39 HCs	Fecal samples	MiSeq sequencing	The gut microbiota of RA patients was characterized by a decreased abundance of *Pholiota*, *Scedosporium*, and *Trichosporon*.
Kitamura et al. (2022) [37]	Observational	*n* = 87 RA	Fecal samples	16s rRNA sequencing	Total bacteria counts were correlated with endotoxin neutralizing capacity (*p* < 0.001) and inversely correlated with serum lipopolysaccharide (*p* < 0.001) and anti-Pg-LPS IgA antibody levels (*p* < 0.001).

**Table 2:** GRADE criteria for risk of bias evaluation. Green: No Risk of Bias; Yellow: Maybe Risk of Bias.

Reference	Methodological Quality	Directness of Evidence	Heterogeneity	Precision of Effect Estimates	Publication Bias	Level of Evidence	Recommendation for Use
Chen et al. (2016) [21]						Moderate	High
Scher et al. 2013 [59]						Moderate	High
Pianta et al. (2018) [60]						Moderate	Low+
Yu et al. (2021) [61]						Moderate	High
Kishikawa et al. (2020) [62]						Moderate	High
Alpizar-Rodriguez et al. (2019) [63]						Moderate	Low+
Chen et al. (2021) [64]						Moderate	High
Diamanti et al. (2020) [65]						Moderate	High
Sun et al. (2022) [66]						Moderate	Low+
Kitamura et al. (2022) [37]						Moderate	Low+
